# Supplementary material for: Integrating Reference Intervals into Chimpanzee Welfare Research
Source: Animals (Basel). 2023 Feb 12;13(4):639. doi: 10.3390/ani13040639 (PMC9951686; doi:10.3390/ani13040639)
Supplement: Supplementary file 1 [file animals-13-00639-s001.zip › animals-2156649-supplementary.pdf]

animals

MDPI

**Supplementary Table S1. Ethogram of species-appropriate behaviors for chimpanzees.**

| Behavior | Modifier | Definition |
|---|---|---|
| **Event Behaviors** | | |
| Affiliative Touch | Agent | Individual engages in non-agonistic social behavior not defined elsewhere in the ethogram. For example, quick touches that occur during greetings. |
| Copulation | | Includes any component of a series of sexual behaviors including mounting, being mounted, thrusting, being thrust-mounted, and complete copulation. Ventral surface of one animal may be in contact with dorsal surface of another briefly or for extended period. May occur with or without full penetration. Also includes unsuccessful copulations due to incorrect orientation or unreceptive partner. |
| Sexual Exam | Agent | Visual, oral, or manual inspection of the ano-genital region of another individual. This should not be confused with grooming or manipulation of the anus to obtain feces. |
| Sexual Present | Agent | Animal's posture varies from slight flexion of the arms or turning of the rump toward another to an extreme crouch with all four limbs folded under it, so it is close to the ground. Can occur in response to a solicit from another individual, or without solicitation. Does NOT occur in response to any agonistic behavior. |
| Supplant | Agent | Individual approaches a conspecific and replaces that individual within one meter of the original location. The supplanted individual moves away to allow the focal to take over that location. |
| Yawn | | Involuntary wide opening of the mouth accompanied by deep inhalation. |
| **State Behaviors** | | |
| Aggressive | Contact, Agent | Agent of aggressive behaviors (assailant) that must involve some physical contact between individuals. Includes wrestle, lunge hit, grab, bite, throw, and scratch. May include pilo-erection. |
| Aggressive | Non-Contact, Agent | Agent of aggressive behaviors (assailant) directed to another individual that do not include any physical contact. Includes lunge, rush, and threats. |
| Contact | | Individual physically touches one or more conspecifics. Includes passive contact (e.g. two individuals in contact as they sleep next to each other) and affiliative active contact (e.g. individuals walk in contact). |
| Display | Chimpanzees | Aggressive behavior without any clear and identifiable chimpanzee recipient. May include pilo-erection, and such behaviors as beat on or move inanimate objects, stomp, slap, sway, hoot, chest-beat, or run. |
| Display | Humans | Aggressive behavior without any clear and identifiable human recipient. May include pilo-erection, and such behaviors as beat on or move inanimate objects, stomp, slap, sway, hoot, chest-beat, or run. |
| Feed/Forage | | Individual handles, manipulates or ingests food items such as primate chow, biscuits, fruits, vegetables, natural vegetation. Includes foraging through bedding or other materials in search of desired food items. Does not include ingestion of feces. Individual ingests water. May be from Lixit nipple, ground source, waterfall or standing water pool. Does not include ingestion of urine. |

| | | |
|---|---|---|
| Groom | Social, Mutual/Multiple | Subject and one or more individuals both pick through each other's hair or skin and remove debris with hand and/or mouth. Does not include pull hair. |
| Groom | Self-Directed | Pick through own hair or skin and remove debris with hand and/or mouth. Does not include pull hair or scratch. |
| Groom | Social, Agent | Pick through hair or at skin of another individual and remove debris with hands and/or mouth. Does not include pull hair. |
| Human Interaction | Orientation, Public | Individual maintains gaze directed towards member of the public. Must be maintained for longer than 3 seconds. Ape must be within 1 meter of the glass/public barrier. May include attention to observer, such as beg or play solicitation. |
| Human Interaction | Orientation, Staff | Individual maintains gaze directed towards member of the care staff. Must be maintained for longer than 3 seconds. Ape must be within 1 meter of the glass/mesh barrier. May include beg or play solicitation. |
| Inactive | | Individual is not moving and not active in any other behaviors listed. Includes sleeping. |
| Locomotion | Horizontal | Individual changes location in horizontal space by walking, running, crawling, etc. The change in location must be greater than one body length. Counts as Horizontal if all four limbs touch the ground, even if subject moves on a slope (e.g. down a hill or set of stairs). |
| Locomotion | Vertical | Individual changes location in vertical space by climbing, sliding, jumping, etc. The change in location must be greater than one body length. Counts as Vertical if limbs are on any other substrate (e.g. ropes), even if subject moves horizontally among the ropes. |
| Masturbation | | Using a body part, object, or part of the cage to stimulate own genitals. Be sure to separate this from grooming of the genitals or manipulation of the genitals to obtain feces. |
| Object Manipulation | Enrichment | Individual visually examines or manipulates various non-food enrichment items (e.g. paper, cardboard, towels), or bedding material (e.g. hay, straw). Individual manipulates prepared enrichment food item (e.g. feeder tube). May include pick up, push, examine, pick at, lick, scratch, pull, rip, and/or shake. Prepared enrichment includes any edible or non-edible item that is given to the chimpanzees to stimulate natural activity and may be in their enclosure temporarily (e.g. blankets, feeder pipes, hay/straw) but not permanent elements of their enclosure such as ropes, grass, etc. |
| Object Manipulation | Other | Individual visually examines or manipulates elements of the environmental structures (e.g. walls, floor, cage mesh, windows or ropes). Manipulates natural vegetation. Manipulation of food items is scored as feed/forage, unless those food items are clearly not being ingested (e.g. manipulate banana peel without eating it), May include pick up, push, examine, pick at, lick, scratch, pull, rip, and/or shake. |
| Object Manipulation | Prepared Enrichment | Individual visually examines or manipulates various non-food enrichment items (such as paper, cardboard, towels, etc.) or bedding material (such as hay or straw). Individual manipulates prepared enrichment food item (e.g. feeder tube). May include picking up, pushing, examin- |

| | | |
|---|---|---|
| | | ing, picking at, licking, scratching, pulling, ripping, and/or shaking. Prepared enrichment includes any edible or non-edible item that is given to the chimpanzees to stimulate natural activity and may be built into their enclosure (e.g. blankets, feeder pipes, hay/straw) but not elements of their enclosure such as ropes, grass, etc. |
| Play | Social | Non-aggressive interactions involving two or more animals. Never accompanied by pilo-erection or agonism; may be accompanied by play-face and/or laugh. Includes rough-and-tumble play (fast-paced, vigorous locomotion, wrestle, hit, pull, chase, bite, etc.), quiet play (slower-paced, gentle-tickling, finger and toe manipulation, etc.) and also includes social play initiation. |
| Play | Solitary | Individual may play alone quietly with hands, fingers, and toes, other body parts, or an object may be handled and be the focus of play. The individual may toss, hold, wear, carry, chew or make contact with the object while making playful movements. May be either boisterous or quiet. May also include active play involving swing, dangle, leap, somersaults, run, gambol, pirouette, and bounce. Vigorous locomotion or rotation of the whole body or its parts is typical. |
| Scratch | Gentle | Rake fingernails over own skin; smaller movements of hand. |
| Scratch | Rough | Rake fingernails over own skin; larger sweeping scratching involving arm movement. |
| Submission | Agent | Agent of submissive behavior, includes crouch, bob, flee, avoid, fear grimace, bared-teeth scream and pant-grunt towards another individual. |
